# Bilateral One-Stage Revision of Infected Total Hip Arthroplasties: Report of Two Cases and Management of Antibiotic Therapy

**DOI:** 10.1155/2016/3621749

**Published:** 2016-01-19

**Authors:** Thomas Pommepuy, Adrien Lons, Kevin Benad, Eric Beltrand, Eric Senneville, Henri Migaud

**Affiliations:** ^1^University of Lille, 59000 Lille, France; ^2^Orthopaedic Department, Lille University Hospital, rue Emile-Laine, 59037 Lille, France; ^3^Northwest Reference Center for Osteoarticular Infections (CRIOAC-G4 Lille-Tourcoing), Lille University Hospital, rue Emile-Laine, 59037 Lille, France; ^4^Department of Infectious Diseases, Gustave Dron Hospital of Tourcoing, rue du Président Coty, 59208 Tourcoing, France; ^5^Orthopaedic Department, Tourcoing Hospital, rue du Président Coty, 59208 Tourcoing, France

## Abstract

Recommendations for the management of chronic and bilateral total hip arthroplasty (THA) infection are lacking. However, this type of infection involves medical problems concerning the management of the antibiotic therapy. We report two cases of such infections operated as one-stage revision. For each case, both hips were infected with the same bacteria (*Staphylococcus caprae* for one patient and methicillin-sensitive* Staphylococcus aureus* for the other). The probabilistic antibiotic treatment started during the first side (after harvesting intraoperative samples) did not prevent the culture of the bacteriologic harvested during the intervention of the second side. Cultures were positive for the same bacteria for both sides in the two cases presented herein. After results of intraoperative cultures, patients received culture-guided antibiotic therapy for three months and were considered cured at the end of a two-year follow-up. Our results suggest one-stage bilateral change of infected THA is a viable option and that early intraoperative antibiotic, started during the first-side exchange, does not jeopardize microbiological documentation of the second side. This work brings indirect arguments, in favor of the use of prophylactic antibiotics during revision of infected THA.

## 1. Introduction

Total hip arthroplasty (THA) infections occur in 0.5 to 3% of patients, and may account for a third of reoperations and require multidisciplinary management [1–3]. Bilateral infections remain sporadic without clear recommendations. The large study from the Société Française d'Orthopédie-Traumatologie (SOFCOT) reported only 3 bilateral out of 691 THA infections and recommended a two-stage strategy, but no guideline was given for the antibiotic therapy in such situations [4]. This issue was not addressed in the document of the recent International Consensus Meeting on Prosthetic Joint Infections (ICMPJI) [5]. It is largely admitted that a microbiological documentation is essential to manage such chronic infections [3–5]. Choosing a one-stage approach with bilateral replacement in the same session requires intraoperative administration of antibiotics before correcting the second side which may lead to false negative bacterial cultures on the second side. [3–6]. Facing two cases of bilateral infection we made the choice of one-stage strategy and we started probabilistic antibiotics intraoperatively for the first side before the change of the second side. We found it interesting to study these two cases to evaluate the influence of antibiotics on the culture of intraoperative samples and so to argument for prophylactic antibiotics during revision of infected THA.

## 2. Observation

**Figure 1:**
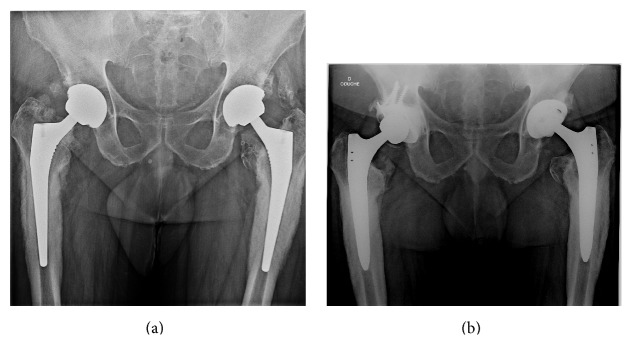
(a) Preoperative AP view case 1 showing typical features of intense femoral periosteal reaction suggesting pain was related to infection. (b) Case  1 AP view at 3 years of follow-up showing regression of periosteal reaction.

The first case was a sixty-year-old male, with an American Society of Anesthesiology (ASA) score of 2 and history of bilateral arthroplasty in 2009 and 2010 done at another institution. He was refereed to our center in 2011 for bilateral hip pain and was suspected of presenting chronic infection, because of radiological signs of intense femoral periosteal reaction (Figure 1(a)). Joint aspiration confirmed the diagnosis, identifying* Staphylococcus caprae* in both hips. The patient underwent a bilateral change in one stage with the administration of broad-spectrum empiric antibiotic therapy as soon as surgical samples were taken on the first side. Replacement of the second side was conducted with the same protocol. Microbiological cultures of both hips were positive with the same bacteria (*Staphylococcus caprae* sensitive to methicillin) found on four samples out of six, on standard and Rosenow's growth medium enriched cultures [7] (Table 1).

The second case was a sixty-year-old female, with an ASA score of 2 and history of bilateral arthroplasty in 2006 and 2009, who consulted in 2012 for left hip pain associated with systemic inflammatory reaction (Table 1). Microbiological cultures of the left hip liquid aspiration were positive with methicillin-sensitive* Staphylococcus aureus* (MSSA). Three weeks later, right hip pain occurred that justified a joint aspiration from the hip in which the same MSSA strain according to the antibiotype was identified. The patient underwent bilateral hip change in one stage according to same surgical and antibiotic therapy protocols as the first case. Cultures of the two hips were positive with the same bacteria (MSSA) on four samples out of six, on standard and Rosenow's growth medium enriched cultures [7].

The pathogens identified during hip aspiration were sensitive to the probabilistic antibiotics. Both cases received the same antibiotic treatment adapted to their antibiograms (rifampicin + levofloxacin) introduced on day 5 for five days intravenously and then for three months per os regimen. At the end of a three-year (case one) and two-year (case two) follow-up, the functional Oxford score [8] was, respectively, 12/60 and 18/60, their CRP was normal, and periosteal reaction had dropped for case one (Figure 1(b)).

## 3. Discussion

Both cases suggest that it seems possible, with a satisfactory microbiological documentation and therefore culture-guided appropriate antibiotic therapy, to perform bilateral one-stage replacement of infected THA. Former studies were in favor of a two-stage revision to manage bilateral infected hip arthroplasties [4], but repeated surgery and an incapacity period due to two-stage replacement led us to prefer the option of one-stage surgery [3, 5, 6].

In the two cases presented above, there are some practical problems. Firstly, the one-stage revision option implies potential surgical risks. However, our patients had low morbidity with an ASA score of 2 and our surgery protocol was strict (change of all prosthesis components and intensive debridement of the surgical site). One-stage strategy allows sterilizing all septic sites and reduces cost (a single hospitalization and antibiotics therapy, a shorter nonworking period) and faster rehabilitation. Secondly, we believe there is a risk of contamination of the nonreplaced side, when the replacement is unilateral. Thirdly, the probabilistic antibiotic therapy could have resulted in false negative culture of the second hip, though it was not reported in our cases. The two cases of the present study advocate the use of antibiotic prophylaxis in case of THA revision for infection [6, 9]. It is however difficult to definitely conclude on this last point as the microbiological documentation is of paramount importance in the management of prosthetic joint infections [5, 6, 9].

## Figures and Tables

**Table 1:** Demographic, microbiological, and therapeutic data.

	Case 1		Case 2	
	Left hip	Right hip	Left hip	Right hip
Date of index surgery	2009	2010	2006	2009
Indication for THA	Primary osteoarthritis			
ASA score	2		2	
CRP level at diagnosis (*N* < 5 mg/L)	18 mg/L		81 mg/L	
White blood cells count (*N* = 4000–10000/mm^3^)	11700/mm^3^		12500/mm^3^	
Neutrophil polynuclear cells count (*N* = 2000–8000/mm^3^)	9360/mm^3^		9850/mm^3^	
Date of joint aspiration	02/11/2011	02/11/2011	02/07/2012	14/07/2012
Bacteria at culture	*Staphylococcus caprae* sensitive to methicillin (coagulase negative)		Methicillin-sensitive *Staphylococcus aureus* (MSSA)	
Date of 1-stage bilateral revision	21/11/2011		06/08/2012	
Delay from probabilistic ATB to sample collection in the second hip	180 minutes		180 minutes	
Probabilistic ATB	Cefepime + daptomycin		Cefotaxime + vancomycin	
Culture-guided ATB debuted intravenously on day 5 for 5 days and then switched to oral for 10 weeks	Rifampicin + levofloxacin		Rifampicin + levofloxacin	
Positivity of culture samples on the second side	Same bacteria 4 out of 6 samples (3 Rosenow's broth and 1 standard)		Same bacteria 4 out of 6 samples (2 Rosenow's broth and 2 standard)	
CRP at the time of withdrawal of antibiotic therapy (*N* < 5 mg/L)	5 mg/L		3 mg/L	
Intraoperative bleeding	1200 cc		1600 cc	
Posttreatment follow-up duration	36 months		24 months	

ATB: antibiotics therapy; CRP: C-reactive protein.
